# Editorial

**Published:** 1870-11

**Authors:** 


### Editorial.

### *A Whisper in Ascalon.*

The Albany dailies are jubilant over the admission to a chair in their Medical College, of an avowed Homœopathist, who is not only a Professor, but the President of the Faculty. The *Evening Journal* of that city discourseth thus:

> "It is, indeed, gratifying, to know that the barriers which have hitherto divided the two schools of medicine are being removed, and to see our college taking the initiatory step towards such a desirable achievement. We believe this is the only allopathic medical institution in this country that possesses views sufficiently liberal to allow any of the chairs to be filled by men who firmly and practically believe in the homœopathic doctrine. It is also pleasant to know that several of the trustees of the college are firm believers in homœopathy.

The *Sunday Morning Press* is less hopeful as to this sign of the times, and lucubrates:

> "We remember some of the professors of this institution making vigorous war against homœopathy, and now we find these same men associated in the institution with a professor who is a homœopath. This astonishing inconsistency is reasonably explainable only on the theory that the Faculty believed when they secured the appointment of Prof. Harris, that it would encourage the patronage of the homœopaths.
>
> "Our medical brethren seem to us to have a chronic disposition to do things in a way a little different from anybody else. Thus, while the clergy and the lawyers have no peculiar laws to govern their respective professions, but yet effectually call their members to account when they overstep the bounds of propriety, the medical profession, on the other hand, establish a long series of laws (their medical ethics) to guide the conduct of its members, apparently having little confidence in their good sense and discretion, and these laws they by no means observe. Thus, the medical profession, unlike the other professions, has a vital law which plainly forbids advertising, and all doctors are supposed scrupulously to abstain from having their names appear in the public prints. But, in fact, the names of medical men are very frequently seen in the newspapers, as every one in this city will testify. Consistency is said to be a jewel, and we believe it to be so, as well in the medical profession as out of it. If the college is allopathic or if it is homœopathic, let it stick throughout to the principles which it professes."

We have seen no denial of this appointment as yet, by the members of the Albany Faculty, or any explanation of the circumstances which, unexplained, of course, put them beyond the pale of legitimate medical association. Now let them open their doors to women, give free tickets to one student from each congressional district, appoint a professor of Free Love and Spiritualism, and the eloquent faculty will no longer be obliged to face a beggarly account of empty benches—however void the heads of the occupants.

### *Fire-Proof Buildings.*

Edwin May, Esq., of Indianapolis, is evidently on the borders of a "glorious summer," as he is engaged in the philanthropic work of developing an improved and cheap mode of rendering buildings fire-proof or non-combustible. The plan consists essentially in the use of iron lath, and also the use of sheet iron, with mortar on top, for a floor lining, which process also deadens sound, and bids defiance to rats. His method should be generally known, and thus one terrible outlet of human life be blockaded.

### *New Journal.*

*The Medical Times; a Semi-Monthly Journal of Medical and Surgical Science.* Published on the 1st and 15th of each month, by J. Lippincott & Co., Philadelphia.

The initial No. bears date Oct. 1st, and promises fairly for the future, at $4 a year. The Prospectus announces a hundred or more regular contributors among the "first families" of the profession. We sincerely trust that the *promising* gentlemen will really contribute. But our own experience is that the promise is, in the majority of cases, the *vox et preterea nihil.* We suggest that the Messrs. Lippincott & Co. put down the names as "Associate Editors," and thus secure at least an article from each.

The matter is copy-righted, or we might be tempted to give our readers a few specimens of its contents. We are comforted by the reflection that a specimen copy may be obtained by enclosing 20 cents of postal currency to the enterprising publishers as above indicated.

It is to be noted that the *Reporter* still continues its issue.

***Indian Medicine.***

From a paper read before the *Orleans County* (Vt.) *Society of Natural Sciences*, by the Rev. T. E. Ranney, who was a missionary three years among the Pawnees, and among the Cherokees fourteen years, we extract the following:

"As to their knowledge of medicine they really have as little as of religion. We are invited to visit a lodge, where one is sick with a fever; we are white men and it is supposed, of course, by them that we can give medicine to cure the sick. But our inquiries are now to ascertain what they would do. We have seen many a handbill recommending various medicines, because it is said it was in use by the Indians, who are supposed by many to understand all medicine and to be able to heal all the sicknesses of the people. Nothing could be a greater evidence of quackery. I do not profess to know that all the tribes of Indians were as ignorant of these things as were the Pawnees, and yet I know nothing that would indicate that any knew more. If the knowledge which the Pawnees had acquired from the whites was taken away, there would be none left. Their medicine, like their religion, is but a bundle of superstition, consisting of charms and jugglery. They are easily imposed upon and made to believe in almost anything hidden and mysterious. They can be made to believe that a peculiar medicine is beneficial, as many in civilized life can; they do not, so far as I could learn, administer any medicine to affect the system. It is doubtful whether they know of anything that would act as a cathartic. For an emetic they seemed to know that tepid or warm water would promote vomiting, but it is doubtful whether they had not learned this from the whites. Some of them could at times produce vomiting by the use of a feather run down the throat. It was not known that the Pawnees ever drew blood intentionally as a remedy. Some of the neighboring tribes did, but it is probable they learned to do so from the whites. Indeed the Pawnees had learned that the whites sometimes did, and supposed it to be a panacea, and if they were ever ill when white men were about who would bleed, they were wont to resort to them with the request to be bled. I frequently saw the manner of cutting themselves practiced by two other tribes, the Omahas and Otoes, to produce blood. They usually bleed somewhere about the head, and to do it shave the hair from the part, often on the temples, sometimes on the top of the head, and then take a large knife and hack the skin up in a horrid manner over a spot perhaps as large as a dollar, and then place over the wound so made the larger end of a cow's horn, and with the mouth and lungs exhaust the air at the other end so as to cause the blood to run, till they have accomplished their object and removed the seat of disease. It was thus these tribes practiced in removing fevers. But not so with the Pawnees. Their doctors would take a different course; they would puff and blow all over the patient with the mouth, in order to cool off the fever, and instead of dieting according to the rules of our physicians they would set before the patient all the tempting viands in their reach. Their philosophy is that while he can eat he will live, and if he does not eat his allowance he will die. Hence it seemed to be almost useless to give medicine to a Pawnee sick with a fever. If the fever is once broken, and the patient begins to recover, his appetite comes like an armed man, and it is useless to tell him he must not gratify that appetite. His maxim is, eat or die, and the *fact* generally is, in such a case, eat *and die.* Their eating is not such as is required to sustain a weakened frame, but such as a sickly appetite demands. Perhaps their practice in cases of fever is most erroneous, but we saw more of this because " chills and fevers " were first introduced among them when we were there. But they did not seem to understand the nature of any disease. They can perform no surgical operation with safety. In cases of accident they cannot take up an artery. Their diseases in their simple life are all of a simple kind, and they seem to have no complex diseases among them. Their practice at an *accouchment* is simply the rattling of a gourd, a child's rattle. I have lately seen a medicine advertised to cure the toothache, which was said to have come from these same Pawnees. A more contemptible imposition could not well be practiced, as they were never subject to any disease of the teeth. An anodyne for the toothache or a vermifuge obtained from them would be alike worthless, they having no use for either."

***Case Book.***

E. P. Stevens & Co., of this city, send us " Shaw's Register and Photographic Case Book," which fills a want we have long experienced. By its use a history of each case treated can be preserved in systematic order, and can be written up for publication or other use with great facility. The best memory will be insufficient to recall in every instance the details of diagnosis and treatment in cases presented perhaps months previously, and a brief reference, which this book provides for, will freshen the whole at once. A portion of the book is prepared for use in preserving photographs of surgical affections, etc. It is of convenient size, and scarcely more expensive than the same amount of paper and binding for an ordinary account book. We notice the book not for the advantage of the publisher, of whom we know nothing, but in the hope that some of our readers will take the hint, make records of their cases, and then publish those worthy in the *Journal.*

***Unhappy Diagnoses.***

It is said that a somewhat noted specialist of this city, who has a trick of disparagement for all non-specialists, recently diagnosed a sero-cystic tumor of the breast as cancer, and attempted its rêmoval as such. In another case, he made an exploratory abdominal incision, *plunged a trochar into a tumor which yielded blood only*, and then by the large incision attempted to remove the tumor which was agglutinated in every part by cancerous deposit to the large and small intestines, etc., etc. *He* recovered.

Yet this man ventures to insult the family physicians of parties who apply to him for examination. It is hoped a glimpse of the rods in pickle for him, will teach him better manners, or, at least, discretion. *Verb. sap.*

***The Western Monthly, for November, 1870.***

CONTENTS: William Cullen Bryant, by James Grant Wilson; Did He Dream It? by Josephine Clifford; the Indian Territory, by Milton W. Reynolds; Public Opinion in Politics, by D. H. Wheeler; Sentenced and Shot, by R. S. Sheppard; Indian Summer, by B. Hathaway; New Constitution of Illinois; Caving In, by H. R. Haines; A Ride through Kauai, by J. T. Meagher; A Western Journalist, by L. D. Ingersoll; Mirage, by Edgar Fawcett; Of Books—their Makers and Readers; by Henry Boss; Who was Souvreigne? by L. A. Roberts; Reviews of Books; Chit Chat. The Lakeside Publishing Company, Publishers, Chicago, Ill.

We are happy to chronicle the reappearance of this valuable and popular Magazine. Its October forms were destroyed in the great fire of September last, but it has arisen from the ashes improved and strengthened. It is now among the best of the literary monthlies, and well worthy of the patronage of Western readers.

***The Prescription Record.***

S. W. Butler, M.D., editor of the *Reporter*, at Philadelphia, sends us a little volume adapted for brief record of the date, symptoms in each case prescribed for—a prescription blank, and blank for its duplicate on each leaf. There are about 400 leaves. It is a very convenient arrangement for rapid record of cases, and it should be on the table of every physician's office, and is not too large to be carried in the pocket. The prescription blanks are punched around so that each when written on it can be easily torn out. Price $1.00. Address, as above, 117 S. Seventh street, Philadelphia.

***Rush Medical College.***

The opening exercises of this institution took place Wednesday evening, the 28th of September. The introductory lecture to the course was given by D. A. Morse, M.D., Lecturer on Legal Medicine and Insanity. The annual course of lectures is now in successful progress before a large and intelligent class.

***Who ?***

A correspondent wishes to know who punched a sound through the walls of a Fond du Lac man's bladder, recently, whilst searching for calculus. All we know about it is, that "somebody blundered," and the man died.

